# The Atlanta Medical Society

**Published:** 1882-05

**Authors:** 


### Societies.

### THE ATLANTA MEDICAL SOCIETY.

*Reported for the Atlanta Medical Rejister.*

MARCH, 18, 1882.

Dr. F. H. O'Brien reported a case of fracture of the radius from inordinate muscular action, with the following history:

About the middle of January, Mr. M——, aged 35 years, applied to him and stated that three days before, while assisting a drayman in lifting a heavy weight, he felt something give way in his wrist, and thought he had sprained it. He however continued the use of the arm, and spent almost the entire two days following the injury in writing and whittling. When he applied for advice he was thoroughly convinced that his arm was broken, having noticed some deformity and hearing crepitation at point of injury. On examination, he found an oblique fracture about three inches above the inferior extremity of the right radius. The arm was put up in ordinary plaster splints. The case was reported because he thought it must be an exceedingly rare occurrence for fracture of the radius to be produced by excessive muscular action. He believed that the biceps pulling upon the radius, and the supinator longus, depressing the inferior extremity of the bone, were the muscles that played the most important part in producing the fracture.

Dr. O'Brien also reported a case of gastritis in an infant, caused by nursing the mother while she was in a state of great excitement, as follows:

On the 7th of January last, Mrs. ——— brought to his office her infant son, aged five months, who presented the following appearance: Great emaciation and pallor; the eyes sunken, and the expression that of an old person; temperature 100½°; pulse exceedingly feeble and too rapid to be accurately counted. The bones of the extremities seemed to be covered only by skin, the muscles having wasted to such an extent. The abdomen was distended, and the epigastric region tender on pressure. It was with difficulty that the child could breathe through the nose, owing to the accumulation of mucus, and it had some little cough. The mother made the following statement: When the child was about five weeks old, she nursed it immediately after returning from the death-bed of her sister, while she was in a state of great emotional excitement. The child, who up to that time had been in perfect health, was seized with violent vomiting and purging immediately after nursing. The physician, who treated it at that time and up to the time she brought it to him, pronounced it gastro-enteritis, which he thinks was the correct diagnosis, but when presented to him, he thinks the stomach was the organ principally affected. The infant showed evidence of intense thirst by eagerly taking anything in a fluid form offered it. The tongue presented a flabby appearance, being dry with red edges. About the time the child began to improve from the acute attack, and as the mother thought, was getting well rapidly, it developed thrush, and very soon the vomiting became as prominent a symptom as before, and remained so up to the time he saw it. The child was having three or four loose stools daily, the character of which alternated between greenish mucus and watery clay-colored discharges. His diagnosis then was chronic gastritis, and to some extent probably follicular. The prognosis was grave.

The treatment for the first two weeks consisted of a powder containing small quantities of cerium oxalate, pepsine and mild chloride of mercury, given every four hours. The diet recommended was milk diluted with water. The vomiting disappeared after the first twelve hours of treatment, and did not reappear to any extent for the next two weeks. He concluded then that the milk diet could be improved upon by including with the milk a preparation known and sold under the name of Imperial granum, which, experience teaches him, is an excellent food for infants in gastro-enteric troubles. About the end of the first month he put the child on a mixture containing small quantities of phosphorous, iron, quinia and strychnia. The child steadily improved, and was dismissed the latter part of March, presenting the appearance of a healthy infant; and, when caution is used in the diet, seems to relish and digest its food.

He would state that he was not fond of prescribing the infant foods the market is so crowded with, not having used any other than that above mentioned.

He thinks the prime remedy, in the beginning, was the cerium oxalate, as the ordinary remedies, such as calomel, bismuth, pepsine, etc, had already been frequently used.

Dr. Jno. Thad. Johnson has used oxalate of cerium in gastric troubles both in children and adults. Usual doses to young children one grain, to adults five to ten grains every two hours. The results from the remedy in his hands have been good. He reported a case, several years ago, of gastritis, consequent upon the ingestion of six or seven drachms of chloroform, in which he administered the remedy—five grains every two hours—with wonderfully good effect. He gives the powder dry, followed by a small quantity of water. He is not as credulous as some concerning the action of remedies, but has confidence in this.

Dr. O'Brien considers it also a valuable remedy in acute alcoholism and in delirium tremens. It seems to relieve the cerebral, as well as the gastric symptoms pertaining to these disorders. Uses it sometimes in doses of fifteen or twenty grains, repeated every six hours.

Dr. Jas. B. Baird said one of the cases reported by Dr. O'Brien represented a class of cases of grave importance, known generally as cholera infantum, which occasion a heavy mortality in the warm months among infants and young children. He expressed the positive conviction that most cases are the result of, and many deaths are due to, improper food. The heat of the weather and unsanitary surroundings doubtless act as predisposing causes by depressing the nervous power of the child, and other sources of irritation may operate in the same way, but the exciting cause of these disorders is, perhaps, in most cases, traceable to injudicious feeding.

He believes the only proper food—the only physiological food—for children, until they get sixteen teeth, is healthy mothers' milk, or the best substitute that can be obtained—cows' milk, carefully chosen and properly prepared, according to the physical condition and the age of the subject. He denounced farinaceous preparations and the so-called "infants' food" so successfully palmed off upon the people and the profession by enterprising manufacturers, and declared them, one and all, to be unnecessary and actually hurtful. Some children, he is aware, seem to thrive upon these preparations. The milk with which they are usually mixed is the saving element, and nutrition goes on, not on account of, but in spite of the farinaceous ingredients. He expressed the belief that many cases of dyspepsia, which prove so troublesome and intractable, in youth and adult life, have their origin in improper feeding during the first year or two of life. Much can be said on this interesting topic, but time suffices merely to hint at a few practical points.

As to treatment, he thinks after correcting errors of diet and providing suitable nourishment, which is the principal thing, that a digestive tonic is often of service; for this purpose he likes pepsine or lactopeptine, and subnitrate of bismuth.

This treatment generally meets his expectations. He does not employ astringents and opium, except rarely, to meet some urgent symptom.

When milk is used it is frequently insufficiently diluted. This is a very important point. It should sometimes be diluted to the extent of three or four parts of water to one of very rich milk.

Dr. O'Brien likes some of the preparations of infants food, especially barley. Mixed with milk they have yielded him good results.

Dr. W. S. Armstrong remarked that he had had no experience with the so-called foods. He never used or indorsed them; approves of nothing for young children outside of milk. Has known of so much trouble resulting from "foods" etc., that it has never occurred to him to try them. The creator has furnished milk as nourishment, and no teeth to masticate solid food. Nature is a safe guide. He always protests against the use of anything but milk; if cows or goats milk it should be properly diluted. Mothers are frequently hard to manage in reference to this subject, and sometimes think, by withholding other forms of food, that they are starving their child. Approves of the use of pepsine, lactopeptine ingluvin, etc.

Dr. Jno. Thad. Johnson indorsed almost entirely the words that had been uttered by the last two speakers; condemned all food for young children save mothers milk, or properly prepared cows milk. Has used the condensed milk occasionally with satisfactory results. Has not much confidence in pepsine, bismuth and such preparations. His experience with condensed milk has been more favorable than his reasoning, *a priori*, would lead him to believe possible. The only test of quality is a trial and observance of effects. It should be sufficiently diluted.

Dr. Baird reported an interesting case of syphilitic paraplegia. The nature of the trouble had not been recognized when the young man, aged about twenty-two years, was referred to him. Both legs were useless, and were the seat of considerable pain; bowels obstinately constipated; bladder partially paralyzed; great pain in the lumbar region. This was substantially the history of the case.

A suspicious eruption on the back and spots here and there over the body and limbs revealed the real cause. Anti-syphililic treatment activily pushed, sufficed, in a few weeks, to effect relief of his disability. The eruption, the constipation and the difficult urination disappeared, and he was able to walk anywhere in the city with only the aid of a light cane.

He mentioned what he considered a practical point in connection with the administration of large doses of the iodide of potassium, namely, giving it just before meals in an alkaline solution. He uses vichy water as a vehicle, or, when economy is to be observed, a pinch of bicarbonate of soda in a glass of well or cistern water will do admirably as a substitute. Patients stand, without discomfort, large doses of the drug administered in this way, when their stomachs rebel against its use in in tonic solutions taken, in the customary manner, after eating.

Dr. Armstrong recalled the history of a case that was prostrated by three different attacks of syphilitic paralysis. He had loss of speech and convulsions. Anti-syphilitic treatment in every instance afforded prompt relief, and it was the omission of the remedies that caused the relapse or the return of the paralytic symptoms.

The first attack occurred about eight years ago. Since the last seizure he has married, but continued the treatment all the time—two years—until very recently, when he determined to abandon it. The almost immediate result was another convulsion. He was on mixed treatment —mercury and iodide of potassium, and tonics.

